# Discovery of Radioiodinated Monomeric Anthraquinones as a Novel Class of Necrosis Avid Agents for Early Imaging of Necrotic Myocardium

**DOI:** 10.1038/srep21341

**Published:** 2016-02-16

**Authors:** Qin Wang, Shengwei Yang, Cuihua Jiang, Jindian Li, Cong Wang, Linwei Chen, Qiaomei Jin, Shaoli Song, Yuanbo Feng, Yicheng Ni, Jian Zhang, Zhiqi Yin

**Affiliations:** 1 Affiliated Hospital of Integrated Traditional Chinese and Western Medicine, Nanjing University of Chinese Medicine, Nanjing 210028, China; 2 Laboratories of Translational Medicine, Jiangsu Province Academy of Traditional Chinese Medicine, Nanjing 210028, China; 3 Department of Natural Medicinal Chemistry & National Center of Drug Screening, China Pharmaceutical University, Nanjing 210009, China; 4 Department of Nuclear Medicine, Renji Hospital, Shanghai Jiaotong University, School of Medicine, Shanghai 200127, China; 5 Theragnostic Laboratory, Campus Gasthuisberg, KU Leuven, 3000 Leuven, Belgium

## Abstract

Assessment of myocardial viability is deemed necessary to aid in clinical decision making whether to recommend revascularization therapy for patients with myocardial infarction (MI). Dianthraquinones such as hypericin (Hyp) selectively accumulate in necrotic myocardium, but were unsuitable for early imaging after administration to assess myocardial viability. Since dianthraquinones can be composed by coupling two molecules of monomeric anthraquinone and the active center can be found by splitting chemical structure, we propose that monomeric anthraquinones may be effective functional groups for necrosis targetability. In this study, eight radioiodinated monomeric anthraquinones were evaluated as novel necrosis avid agents (NAAs) for imaging of necrotic myocardium. All ^131^I-anthraquinones showed high affinity to necrotic tissues and ^131^I-rhein emerged as the most promising compound. Infarcts were visualized on SPECT/CT images at 6 h after injection of ^131^I-rhein, which was earlier than that with ^131^I-Hyp. Moreover, ^131^I-rhein showed satisfactory heart-to-blood, heart-to-liver and heart-to-lung ratios for obtaining images of good diagnostic quality. ^131^I-rhein was a more promising “hot spot imaging” tracer for earlier visualization of necrotic myocardium than ^131^I-Hyp, which supported further development of radiopharmaceuticals based on rhein for SPECT/CT (^123^I and ^99m^Tc) or PET/CT imaging (^18^F and ^124^I) of myocardial necrosis.

Coronary artery disease (CAD) is a major cause of mortality worldwide, resulting in 8.14 million deaths (16.8%) in 2013^1^. CAD occurs when the arteries that supply blood to myocardium become hardened and narrowed. Timely revascularization as the most effective therapy for patients may restore adequate blood flow, improve cardiac function, prevent further deterioration in left ventricular function and reduce the risk of sudden death^2^,^3^. Although revascularization has served to reduce the overall mortality from CAD, it is not always appropriate for all patients. The Occluded Artery Trial (OAT) is a multicenter, international and randomized study that designed to test routine coronary intervention after myocardial infarction (MI). The results of OAT show that revascularization has no curative benefit for some patients with little reversibly viable myocardium in infarcted areas. Re-infarctions (approximately 10% of re-infarctions being rapidly fatal) and serious reperfusion injury tend to happen at a higher rate in those patients randomized to myocardial revascularization^4^,^5^,^6^,^7^. Therefore, the assessment of myocardial viability is deemed necessary to aid in revascularization therapy and to improve the outcome of patients with CAD.

Non-invasive cardiac imaging plays an important role in anatomical and functional evaluations of MI. In clinical practice, most tracers used for the assessment of myocardial viability are usually through two main physiologic mechanisms: myocardial perfusion and cellular metabolism in viable areas^8^. ^18^F-FDG is a gold standard for the evaluation of viability, which is based on segmental differences in perfusion and metabolism to differentiate normal, dysfunctional but viable and necrotic myocardium. However, the results are likely to be false negatives and positives due to the uptake of ^18^F-FDG are easily influenced by the glucose concentration, insulin level and metabolism of individuals^9^.

The imaging of necrotic myocardium may enable the evaluation of MI by assuring the proportions of irreversibly infarcted versus jeopardized but still viable viability myocardium^10^,^11^. Relative to programmed cell death or apoptosis which is detectable with annexin V tracers but less relevant to acute MI^12^, necrosis is a common type of cell death and is characterized by irreversible loss of plasma membrane integrity with cells welling and rupture, leading to release of intracellular contents^13^. Some components such as exposed DNA, histones, phosphatidylserine, etc. released from necrotic tissues are potential targets for the identification of necrotic myocardium to assess myocardial viability^13^,^14^. Necrosis avid agents (NAAs) selectively accumulate in necrotic tissues, which could be exploited for “hot spot imaging” of necrotic myocardium to measure non-viable infarcted areas^15^ and to estimate prognosis^16^. Moreover, radioscintigraphic NAAs in trace amount may prevent dysfunctional but viable myocardium from the further damage caused by excessive uptake of contrast agents for other less sensitive imaging modalities^8^. ^99m^Tc-pyrophosphate^17^, ^111^In-antimyosin Fab antibody^18^ and ^99m^Tc-glucarate^19^ have been used to localize and quantify myocardial necrosis in patients. But they have not been widely accepted in the clinic because they cannot fulfill these requirements: high avidity and specificity, rapid localization at the infarct and reasonable duration of scan positivity.

Recently, hypericin (Hyp)^10^, protohypericin^20^ and Sennidin A^21^, which are polyphenolic polycyclic dianthrone compounds (Fig. 1), have been recognized as prominent NAAs. As candidate positive tracers for imaging of necrotic myocardium, they showed high affinity for necrotic tissues after being labeled with radionuclides^10^,^15^,^20^,^21^,^22^,^23^,^24^. However, the long plasma half-life time of radio-labelled Hyp with high blood pool activity *in vivo* would make it unsuitable for early imaging of necrotic myocardium after administration. Although high imaging contrast between infarcted and viable myocardium reached at 9 h after injection^15^, clear visualization of necrotic myocardium was not possible within the clinically relevant time window for thrombolytic therapy (usually within 6 h of the acute event), which is the problem that need to be solved in clinical practice.

In this context, there is a pressing need for finding novel NAAs with the potential for early imaging of necrotic myocardium. Hyp, protohypericin and Sennidin A, all showing specific necrosis affinity, are composed by coupling two molecules of monomeric anthraquinone in different ways^25^,^26^. Based on the evidence that the active center can be found by splitting chemical structure^27^, monomeric anthraquinones may be effective functional groups for necrosis targetability, which share simplified structures and shorter plasma half-life times^28^,^29^,^30^. Hence, we speculated that monomeric anthraquinones may also feature necrosis targetability and could be used for imaging of necrotic myocardium earlier than Hyp.

To validate this hypothesis, we selected eight monomeric anthraquinones (Fig. 2) and labelled them with iodine-131, evaluated the necrosis targetability. We further validated the potential of a lead compound for assessment of myocardial necrosis by SPECT/CT imaging and enzymatic histochemical staining with triphenyltetrazolium chloride (TTC).

## Results

### Chemistry of mono-iodorhein

The structural elucidation of mono-iodorhein was determined by ^1^H, ^13^C NMR and HR-ESI-MS experiments (Supplementary Fig. S1–3), indicating that H-7 on the aromatic ring has been replaced by the iodine atom. ^1^H NMR (300 MHz, DMSO-*d*_*6*_) δ (ppm): 8.36 (d, J = 7.5 Hz, 1H, H-6), 8.14 (s, 1H, H-4), 7.79 (s, 1H, H-2), 7.48 (d, J = 7.8 Hz, 1H, H-5); ^13^C NMR (300 MHz, DMSO-*d*_*6*_) δ (ppm): 191.2 (C-9), 181.0 (C-10), 165.5 (-COOH), 161.3 (C-8), 160.4 (C-1), 146.8 (C-6), 138.5 (C-3); 134.1 (C-4a), 133.3 (C-10a), 124.6 (C-2), 120.6 (C-5), 119.0 (C-4), 118.7 (C-8a), 115.9 (C-9a), 96.3 (C-7). HR-ESI-MS: calculated for [C_15_H_7_O_6_I]: 408.9209 [M−H]^−^; found 408.9215 [M−H]^−^.

### Radiochemistry and *in vitro* stability

The Iodogen coating method was conducted successfully in this study. Radiochemical yields of ^131^I-1-hydroxyanthraquinone, ^131^I-alizarin, ^131^I-danthron, ^131^I-rhein, ^131^I-aloe-emodin, ^131^I-chrysophanol, ^131^I-emodin and ^131^I-physcion were 92.13% ± 0.53%, 97.49% ± 0.47%, 93.93% ± 0.59%, 96.71% ± 0.43%, 96.15% ± 0.46%, 95.01% ± 0.51%, 99.32% ± 0.59% and 94.27% ± 0.42% (without purification) respectively as determined by TLC shown in Supplementary Fig. S4. All ^131^I-anthraquinones turned out to be stable after incubation in rat plasma at 37 °C for 24 h with the data of 87.93% ± 0.45%, 93.17% ± 0.49%, 90.14% ± 0.48%, 93.17% ± 0.38%, 91.90% ± 0.43%, 90.04% ± 0.49%, 98.72% ± 0.46% and 91.06% ± 0.38%, respectively.

### Chemical purity and *in vitro* stability by HPLC

**Figure 3:**
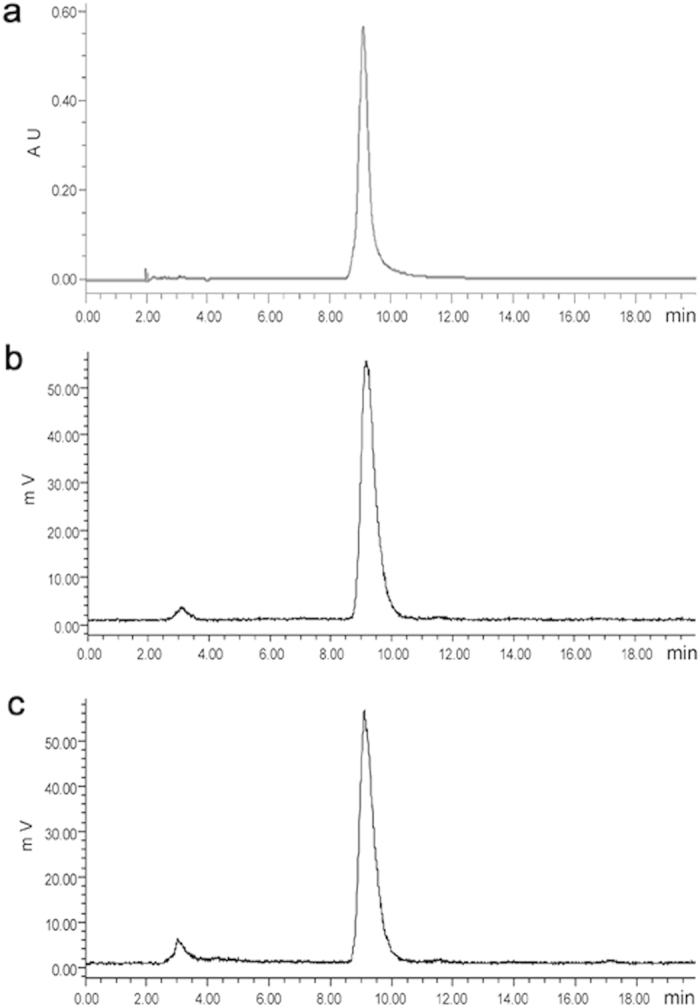
HPLC chromatograms of mono-iodorhein or ^131^I-rhein. (**a**) ultraviolet-chromatogram of mono-iodorhein; (**b**) radiochemical purity of ^131^I-rhein in injection preparation; (**c**) *in vitro* stability of ^131^I-rhein after 24 h incubation in rat serum at 37 °C.

HPLC analysis showed the retention time of mono-iodorhein was 9.08 min (Fig. 3a), which was consistent with that of ^131^I-rhein as 9.14 min (Fig. 3b). Radiochemical purity of ^131^I-rhein was 95.27% ± 0.53% as detected with HPLC by quantifying peaks corresponding to the free ^131^I and ^131^I-rhein. The *in vitro* stability data showed 91.75% ± 0.47% of the intact parent tracer after 24 h incubation in rat serum (Fig. 3c), indicating that ^131^I-rhein was stable in rat plasma till the detection endpoint. The results of radiochemical purity and *in vitro* stability of ^131^I-rhein determined by radio-HPLC were consistent with that by TLC.

### Biodistribution studies of mice

The biodistribution results of all ^131^I-anthraquinones were shown in Supplementary Table S1–2. These tracers showed different necrosis targetability *in vivo* and the uptakes of all ^131^I-anthraquinones by necrotic muscle were significantly higher than that by viable muscle at three time points (*P* < 0.01). The radioactivity ratio of necrotic to viable muscle for each tracer at 24 h post-injection (p.i.) was higher than that at 2 h or 12 h p.i. (*P* < 0.05). Relatively high uptakes of eight ^131^I-anthraquinones were detected in organs/tissues such as necrotic muscle, blood, liver, kidney and stomach while viable muscle and heart showed the lowest uptake at 2 h p.i. These data displayed an obvious clearance of radioactivity from all normal organs within 24 h except for the kidney and liver, indicating that these tracers were mainly metabolized and/or excreted by such dual pathways.

The concentrations of ^131^I-rhein in necrotic vs viable muscle were 4.61 ± 0.39%ID/g vs 0.79 ± 0.08%ID/g, 4.06 ± 0.16%ID/g vs 0.56 ± 0.04%ID/g and 0.74 ± 0.04%ID/g vs 0.06 ± 0.00%ID/g at 2 h, 12 h and 24 h after administration with the corresponding radioactivity ratios from necrotic to viable muscle were 5.84 ± 0.10, 7.26 ± 0.23 and 12.33 ± 0.67, respectively. The necrotic uptake and radioactivity ratio of ^131^I-rhein were the highest than that of other seven tracers at three time points (*P* < 0.01). The clearance of ^131^I-rhein from normal organs was also favorable for imaging purpose.

### Outcomes of TTC staining and autoradiograhy

**Figure 4:**
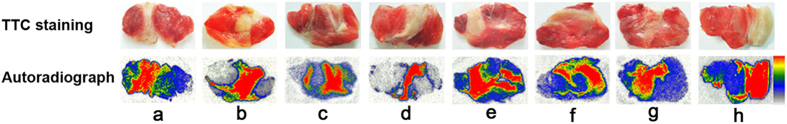
TTC staining images and autoradiographs of eight tracers from mice sacrificed 24 h after administration. On the TTC-stained images, the necrotic muscles remained pale whereas normal muscles were stained brick red. The high radioactivity uptakes (in red) primarily appeared in necrotic regions on autoradiograms, which were in accordance with pale areas on TTC staining images. (**a**–**h**) were represented ^131^I-1-hydroxyanthraquinone, ^131^I-alizarin, ^131^I-danthron, ^131^I-rhein, ^131^I-aloe-emodin, ^131^I-chrysophanol, ^131^I-emodin and ^131^I-physcion, respectively.

Figure 4 displays close matches between TTC stained slices and corresponding autoradiograms from model mice sacrificed at 24 h after administrations of all eight tracers. On the TTC-stained specimens, the necrotic muscles remained pale whereas normal muscles were stained brick red. The high radioactivity uptakes (in red) primarily appeared in necrotic regions on autoradiographs, which were in accordance with pale areas on TTC-staining images. By quantitative autoradiography analyses, the necrotic/viable muscle ratios of ^131^I-1-hydroxyanthraquinone, ^131^I-alizarin, ^131^I-danthron, ^131^I-rhein, ^131^I-aloe-emodin, ^131^I-chrysophanol, ^131^I-emodin and ^131^I-physcion were 9.16 ± 0.78, 9.84 ± 0.89, 9.93 ± 0.83, 17.64 ± 1.28, 11.08 ± 0.91, 10.34 ± 0.86, 9.61 ± 0.72 and 8.14 ± 0.79, respectively. These data were consistent with the results of biodistribution studies. Among these tracers, ^131^I-rhein showed the highest necrotic/viable muscle ratio at 24 h after administration (*P* < 0.05).

The results obtained from biodistribution, TTC staining and autoradiography indicated that all eight ^131^I-anthraquinones selectively accumulated in necrotic muscles and ^131^I-rhein appeared to be the best radiopharmaceutical NAA, which encouraged us to further evaluate it by *in vivo* SPECT/CT imaging in rats with MI.

### Pharmacokinetics study in normal rats

The elimination half-life (t_1/2z_) of ^131^I-rhein was 8.20 ± 0.49 h, which presents a relatively fast clearance from blood circulation. The radioactivity concentration of ^131^I-rhein in blood at 6 h decreased to 23.47% ± 1.91% of peak radioactivity concentration. The major pharmacokinetic parameters are given in Table 1.

### SPECT/CT imaging of rats

**Figure 5:**
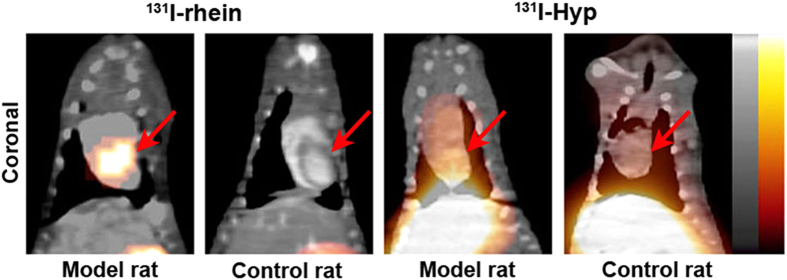
SPECT/CT imaging of ^131^I-rhein or ^131^I-Hyp (as a positive control agent) in rats with reperfused MI or sham operation. SPECT/CT-fused images showed high uptake of ^131^I-rhein in the MI and no obviously high radioactivity in viable myocardium, relatively high radioactivity of ^131^I-Hyp was observed in heart and liver of model rat or sham operation rat. Red arrows indicate the heart.

The *in vivo* SPECT/CT images of rats with reperfused MI or sham operation at 6 h after administration of ^131^I-rhein or ^131^I-Hyp are exemplified in Fig. 5. Relatively high uptake as a hot spot showed up in the heart of the model rat, while no obvious uptake was observed in the heart of the control rat, indicating a selective accumulation of ^131^I-rhein in necrotic myocardium. Prominent activity was seen in stomach likely due to the gastric excretion of the free iodide (^131^I). With ^131^I-Hyp, due to the high blood pool activity at 6 h p.i., relatively high radioactivity was observed in the heart of the model rat and the sham operation rat, which could not distinguish between viable and necrotic myocardium. Moreover, the high activity of ^131^I-Hyp in the liver may compromise the imaging accuracy of necrotic myocardium.

### Biodistribution of ^131^I-rhein and ^131^I-Hyp in model rats

**Figure 6:**
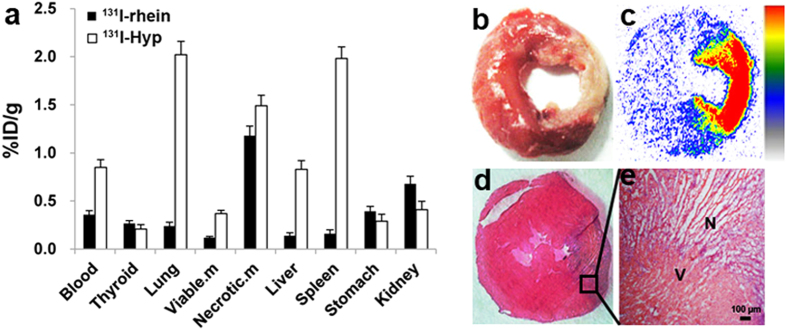
Biodistribution and postmortem analyze of MI in rats. (**a**) Biodistribution of ^131^I-rhein or ^131^I-Hyp at 6 h after administration (n = 5 per tracer). Data are expressed as average percentage of injected dose per gram (%ID/g) ± SD: Viable.m = viable myocardium, Necrotic.m = necrotic myocardium; (**b**–**e**) Postmortem analyze of necrotic and viable myocardium from model rats of ^131^I-rhein: (**b**) TTC staining image of 2 mm thick slice; (**c**) autoradiogram from 50 μm frozen section; (**d**) H&E staining image of 10 μm frozen section; (**e**) H&E-stained microphotograph proved the presence of necrosis: N = necrotic area, V = viable area.

Figure 6a reveals a high and specific radioactivity uptake of ^131^I-rhein in necrotic myocardium at 6 h p.i. The radioactivity concentration of ^131^I-rhein in infarcted myocardium was 1.18 ± 0.03%ID/g, which was about 9.81 ± 0.61 times higher than that in viable myocardium (0.12 ± 0.01%ID/g). Prominent activity was detected in the kidney (0.68 ± 0.04%ID/g), indicating a major renal pathway for this tracer. The radioactivity concentration of ^131^I-rhein in blood, lung and liver were 0.36 ± 0.03%ID/g, 0.24 ± 0.02%ID/g and 0.14 ± 0.01%ID/g, respectively, all were lower than the radioactivity in necrotic myocardium (*P* < 0.01), suggesting ^131^I-rhein as a promising imaging tracer of MI with satisfactory heart-to-blood, heart-to-liver, and heart-to-lung ratios.

When compared with ^131^I-rhein, ^131^I-Hyp had higher viable myocardium uptake (0.37 ± 0.03%ID/g) and the lower necrotic/viable myocardium ratio of 4.03 ± 0.03 at 6 h p.i. (*P* < 0.01). Higher radioactivity concentrations of ^131^I-Hyp were detected in blood (0.85 ± 0.08%ID/g), lung (2.02 ± 0.14%ID/g) and liver (0.83 ± 0.09%ID/g) than that of ^131^I-rhein (*P* < 0.01), which would compromise the imaging of MI at 6 h p.i. These data were consistent with the results of SPECT/CT images.

### Histopathological staining and autoradiography of MI

The histopathological results of ^131^I-rhein from model rats were shown in Fig. 6b–e. On the TTC-stained slice (Fig. 6b), the infarcted myocardium remained pale whereas viable myocardium was stained brick red. The infarcted areas involve the lateral wall of the left ventricle (TTC negative). A homogenous high radioactivity uptake (in red) only occurred in the infarcted areas on autoradiograph (Fig. 6c), which corresponded well to the pale region on TTC stained specimen. Quantitatively, 14-fold higher activity was found in infarcted myocardium than that in viable myocardium. The H&E staining (Fig. 6d) and photomicrograph (Fig. 6e) confirmed the presence of necrosis.

## Discussion

In this study, we identified monomeric anthraquinones as a new class of NAAs, which have simpler structures compared to other recently reported tracers^31^. These novel agents showed prominent targetability to necrotic tissues in animal models. Among these NAAs, ^131^I-rhein emerged as the most promising compound to be an excellent “hot spot imaging” tracer potentially for imaging of necrotic myocardium to deduce myocardial viability. Moreover, the timing for visualizing necrotic myocardium with ^131^I-rhein was earlier than that with ^131^I-Hyp.

We selected mice model of muscular necrosis to screen the necrosis affinity among eight monomeric anthraquinones with radioiodination. All data indicated prominent targetability of eight ^131^I-anthraquinones to necrotic tissues, with highly sustaining necrotic to viable tissues radioactivity ratios and efficient clearance of radioactivity from non-target organs. Furthermore, the substituents in their aromatic ring appear to have impact on their necrosis targetability. The necrotic/normal radioactivity ratios and the necrotic uptake with ^131^I-rhein were the highest than that with other seven tracers, indicating that the carboxyl group at the 3-position might favor the necrosis targetability.

Based on the results above, we selected ^131^I-rhein as a candidate lead compound to explore its imaging potentials in rat model of MI. ^131^I-rhein (t_1/2z_ = 8.20 h) showed a relatively faster clearance from blood circulation than ^131^I-Hyp (t_1/2z_ = 30.48 h)^22^, which facilitated the early visualization of infarcts. In the previous study, images of high contrast between infarcted and viable myocardium were obtained at 9 h after injection of radio-labelled Hyp and visualization of necrotic myocardium was not possible at earlier stage due to high blood pool activity^15^. These results are consistent with this study that ^131^I-Hyp could not cause hot spot imaging of necrotic myocardium at 6 h after administration. By contrast, we obtained good quality images that visualized necrotic myocardium at 6 h after ^131^I-rhein administration. According to the biodistribution data, the radioactive uptakes in blood, liver and lung were much lower than that in infarcted myocardium, suggesting satisfactory heart-to-blood, heart-to-liver and heart-to-lung ratios with ^131^I-rhein in model rats and this may be the major reason for obtaining images of good quality. However, images of ^131^I-Hyp looked poor due to the high uptakes in blood, liver and lung that are near the heart, which may compromise the diagnostic capacity.

If ^131^I-rhein can be used for imaging of necrotic myocardium in patients at 6 h after administration, the 6 h time window presented in this study deems clinically relevant as thrombolytic therapy is routinely performed prior to visualization and sizing of infarcted areas after the patients are admitted to the emergency unit. Rapid time to thrombolysis treatment within 6 h after MI could be an important determinant for the patient survival with reduced mortality and disability^32^,^33^. Clear demarcation of irreversibly infarcted and the ischemic but still viable myocardium (salvageable tissue at risk of infarction) should be obtained before thrombolysis is initiated within 6 h. Therefore, the performance that ^131^I-rhein has demonstrated in this study could allow the rapidly use of interventions for myocardial salvage through imaging of necrotic myocardium to assess myocardial viability, thereby improving the clinical outcomes of treatment.

Some tracers have been used for imaging of myocardial necrosis in patients^34^. Like ^131^I-rhein, most tracers made full use of membrane disruption, the hallmark of necrosis as a target. ^99m^Tc-pyrophosphate was feasible for necrosis imaging at 3 h p.i., but the maximal uptake in the necrotic myocardium occurs at 24–72 hours after infarction^35^. This tracer also required residual blood flow, which prevented uptake in the infarct center for a full picture of necrosis^36^. ^99m^Tc-glucarate has good imaging characteristics of necrotic myocardium, but its use is limited to 9 hours after onset, because the target of this tracer is histone that is quickly washed out from necrotic tissues^19^. In this regard, ^131^I-rhein seems a potential NAA for imaging of necrotic myocardium and needs to be further evaluated in follow-up studies.

Further studies are necessary to determine the earliest time point after radiolabeled rhein administration, at which diagnostically useful images can be obtained. Although iodine-131 emits both β-particles and γ-rays that can be used for diagnosis and therapy of cancer and hyperthyroidism, the beta emission may cause damage to adjacent normal myocardium hence enlarge the infarcted areas. In the present study, we have demonstrated that all ^131^I-anthraquinones showed necrosis targetability at 2 h p.i., so we intend to label rhein with these radionuclides (^18^F, ^99m^Tc, ^123^I) that share shorter half-life times for early imaging of necrotic myocardium in the future. We will use ^18^F-FDG-PET/CT (a viability tracer) to compare with ^131^I-rhein-SPECT/CT (a necrosis avid tracer) to further confirm the potential of ^131^I-rhein for the assessment of myocardial viability in the next study.

These new radioiodinated monomeric anthraquinone tracers showed peculiar affinity to necrotic tissues in animal models. We have successfully validated that ^131^I-rhein possessed the diagnostic potential for earlier assessment of myocardial viability through “hot spot imaging” of infarcted myocardium in model rats than ^131^I-Hyp. We consider that radiolabeled rhein represents a new lead compound, worthy of further research toward the ultimate aim of identifying a clinical candidate suitable for SPECT/CT or PET/CT imaging of necrotic myocardium to assess myocardial viability.

## Methods

### Materials and reagents

Kunming mice (male, 22–26 g) and Sprague-Dawley rats (male, 260–280 g) were provided by the Experimental Animal Center of Academy of Military Medical Sciences. The Institutional Animal Care and Use Committee approved the project. The care and treatment of all animals were maintained in accordance with NIH publication No.85–23 (revised in 1996) on “Principles of laboratory animal care”. Eight anthraquinones were commercially available from Chendu SinoStandards Biological Technology Co., Ltd. (Chendu, China) with purity greater than 97%. Sodium iodide’s (Na^131^I) radionuclidic purity was >99% and specific activity was 370 MBq/mL, which was supplied by HTA Co., Ltd. (Beijing, China).

Reversed-phase high performance liquid chromatography (RP-HPLC) was equipped with Waters 2998 PDA detector, HERM LB500 radiometric detector (Berthold Technologies, Germany), a pump (Waters 2695) and a Alltima C18 column (250 × 4.6 mm, 5 μm, GRACE). The mobile phase was methanol/0.1% phosphoric acid in water (80:20, v/v), the flow rate was 1.0 mL/min and the temperature of column was 30 °C. All solvents used for HPLC analysis were HPLC grade.

### Synthesis of mono-iodorhein

**Figure 7:**
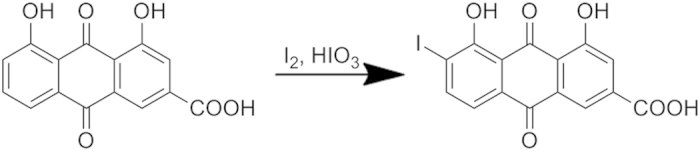
Synthesis of mono-iodorhein.

I_2_ (317 mg, 1.25 mmol) and HIO_3_ (220 mg, 1.25 mmol) were added to the mixture of rhein (284 mg, 1 mmol) in 1,4-dioxane (40 mL) and water (8 mL) (Fig. 7). The reaction mixture was heated to reflux for 24 h before cooling to room temperature and pouring into water (500 mL). The precipitate was collected by vacuum filtration and purified by semipreparative RP-HPLC (methanol: 0.5% three fluorine acetic acid in water =75:25) to give mono-iodorhein (110 mg, 0.27 mmol, 26.85%) as an orange red solid. Its structural elucidation was determined by ^1^H, ^13^C NMR and HR-ESI-MS experiments.

### Radiochemistry and *in vitro* stability

Iodogen (1, 2, 4, 6-tetrachloro-3a, 6a-diphenylglycouracil; Sigma, St. Louis, MO) was dissolved in dichloromethane and deposited on the wall of tubes as a thin film. Radioiodination was initiated by adding dimethylsulfoxide (DMSO) solutions of each anthraquinone (1.25 mg/mL) and Na^131^I solutions (4:1, v/v) into Iodogen-coated tube, adjusting pH with phosphate buffered saline (PBS, pH 7.4). Then these mixtures were shaken and incubated at 45 °C for 2–4 h, and radiochemical purity was determined by thin-layer chromatography (TLC) using Whatman No.1 filter paper and 0.1 M hydrochloric acid as the developing solvent. Then the solvent was evaporated, the paper was cut in half and measured the radioactivity by direct γ-counting (WIZARD1470; PerkinElmer, USA). For obtaining the injectants, these mixtures were diluted with propylene glycol and polyethylene glycol 400 (1:1, v/v), respectively.

*In vitro* stability of eight ^131^I-anthraquinones were tested in rat serum, 0.1 mL injectant was added to 0.9 mL rat serum (1:9, v/v) and incubated at 37 °C. Then radiolabeling stability was determined by TLC using the procedure described above at the time points of 0.5 h, 6 h, 12 h and 24 h, respectively.

### Chemical purity and *in vitro* stability by HPLC

#### Chemical purity of mono-iodorhein or ^131^I-rhein

Chemical purity was detected by HPLC using the procedure described above. The percent of the intact ^131^I-rhein was determined by quantifying peaks corresponding to the intact and degraded products. The assay was repeated three times.

#### *In vitro* stability of ^131^I-rhein

^131^I-rhein was incubated in rat serum at 37 °C for 24 h, and the mixture was mixed with methanol at a ratio of 2:3 and shaken. After centrifuging the mixture at 12000 rpm for 10 min, the supernatant was collected and passed through 0.22-μm pore-size filters (Membrana, Wuppertal, NRW, Germany) for *in vitro* stability analysis by radio-HPLC as described above.

### Animal models of necrosis

All animals were given 0.12% potassium iodide in drinking water from 3 days before the experiment till the end of experiment to protect the thyroid gland from taking up free ^131^I.

#### Muscular necrosis model

Each mouse was intramuscularly injected with 0.1 mL absolute alcohol in the left hind limb to induce muscular necrosis.

#### Myocardial infarction model

Sprague-Dawley rats were intraperitoneally anesthetized with 10% chloral hydrate (3 mL/kg of body weight), then intubated and artificially ventilated with room air using a rodent ventilator. A left thoracotomy was performed along the third and fourth intercostal space, followed by incision of the pericardium to fully expose the heart in thoracic cavity. The proximal left anterior descending coronary artery (LAD) was occluded by a single ligature at 1–2 mm below the junction of the pulmonary conus and the left atrial appendage. In control rats, the suture was passed underneath the LAD without ligation. The chest wall was closed by suturing in layers, with the end of the suture of detachable knot externalized. Sixty minutes after LAD occlusion, pulling the suture end to achieve coronary reperfusion with the rat being intramuscularly injected with 80000 U Penicillin for preventing infection.

### Tracer administration

At least 12 h after recovery from surgery, each model mouse was intravenously administrated with 0.37 MBq of ^131^I-anthraquinones and each rat was intravenously injected with 3.7 MBq of ^131^I-rhein or ^131^I-Hyp for pharmacological evaluations. All animals were intravenously administered with 0.1 ml of 1% Evans blue solution for intravital staining of necrosis that helped to selectively sample bluish necrotic tissues for radioactivity quantification.

### Biodistribution studies in mice

One hundred and twenty model mice were randomly divided into 24 groups for eight tracers (n = 5 per compound and per time point). Mice were sacrificed by cervical dislocation at 2 h, 12 h and 24 h after administration. Necrotic and normal tissues of interest (blood, thyroid, lung, heart, liver, spleen, stomach, kidney, viable/necrotic muscle) were sampled, and wet weighed with the radioactivity measured by a γ-counter. Background radiation and physical decay were corrected during counting. The results were expressed as percentage of the injected dose per gram of tissue (%ID/g). Ratios of radioactivity concentrations between in necrotic and viable muscle were calculated.

### TTC staining and autoradiograhy of normal and necrotic muscles

We further proved the presence of necrotic tissues with enzymatic histochemical staining using TTC on the muscles after biodistribution studies. The necrotic muscles were stained in 2% TTC solution at 37 °C for 15 min and digitally photographed, then cut into 50 μm frozen sections with a cryotome (Shandon FSE, Thermo Fisher Scientific Co., Waltham, MA, USA) for autoradiography. Autoradiographs of these sections were obtained by exposure to a high-performance storage phosphor screen (Cyclone; Canberra-Packard, Ontario, Canada) for 6–48 h and digitally photographed with Optiquant software (Pacard, Packard Meriden, CT, USA). Relative tracer concentrations in necrotic muscles were evaluated by regions of interest analysis and the results were compared with that from the corresponding viable muscles.

### Pharmacokinetics study of ^131^I-rhein in normal rats

For the pharmacokinetic study, six healthy rats were intravenously injected with ^131^I-rhein under anesthesia. The blood samples (10 μL) were collected by means of a tail incision at 5 min, 10 min, 30 min, 1 h, 2 h, 4 h, 6 h, 8 h, 10 h, 12 h and 24 h after administration. The radioactivity of blood samples was measured and the results were expressed as megabecquerel per liter (MBq/L). The blood pharmacokinetic parameters of the radiotracer were analyzed by using the non-compartmental analysis of Drug and Statistics for windows 2.0 software.

### SPECT/CT imaging of rats

The SPECT/CT system (Precedence 6; Philips) consisted of a variable-angle dual-detector with low-energy high-resolution collimators and a multislice spiral CT component optimized for rapid rotation. SPECT/CT imaging was conducted at 6 h after injection of ^131^I-rhein or ^131^I-Hyp (as a positive control agent), MI model rats and sham operation rats were anaesthetized with intraperitoneal injection of 10% chloral hydrate. The SPECT acquisition (128 × 128 matrix, 30 frames) was performed using 6° angular steps in a 25-s time frame. For CT (120 kV, 240 mA, 0.75 s/r), 1 mm slices were obtained. After reconstruction, SPECT images were corrected for attenuation and scatter. Both SPECT and CT axial 1-mm slices were generated using an Astonish bone application package and were transferred to a picture archiving and communication systems after generation of DICOM files. SPECT/CT images were fused using the Syntegra software.

### Biodistribution of model rats

After the scan of SPECT/CT, ten model rats (n = 5 per tracer) were immediately sacrificed by overdose of anesthetic. Necrotic and normal myocardial tissues were collected, weighed and counted as the procedure described above.

### Histopathological staining and autoradiograhy of MI

The isolated hearts from model rats having received ^131^I-rhein were imbedded in a Plexiglas heart matrix by 3% agar solution at 40 °C. A series of 2 mm sections were made in the axial plane. One half of sections were immediately stained in 2% TTC solution at 37 °C for 15 min and digitally photographed, then were cut into 50 μm frozen sections with a cryotome for autoradiography as above. Slices (10 μm) adjacent to those used for TTC staining were selected for hematoxylin-eosin (H&E) staining using standard techniques and digitally photographed, which were then photomicrographed under a bright field.

### Statistical analysis

All quantitative results were expressed as mean ± standard deviation (SD). Between-group or data from tissues of interest were compared by one-way analysis of variance using Student’s *t* test and *P* < 0.05 was considered statistically significant.

## Additional Information

**How to cite this article**: Wang, Q. *et al.* Discovery of Radioiodinated Monomeric Anthraquinones as a Novel Class of Necrosis Avid Agents for Early Imaging of Necrotic Myocardium. *Sci. Rep.* **6**, 21341; doi: 10.1038/srep21341 (2016).

## Supplementary Material

Supplementary Information

## Figures and Tables

**Figure 1:**
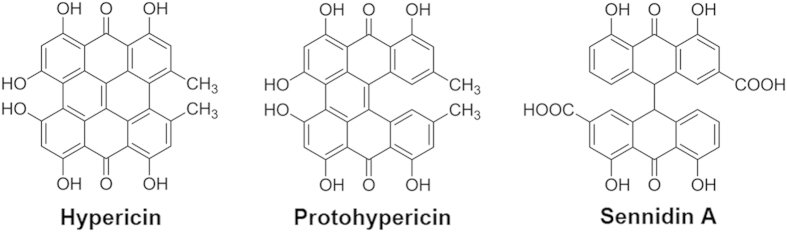
Chemical structures of reference compounds.

**Figure 2:**
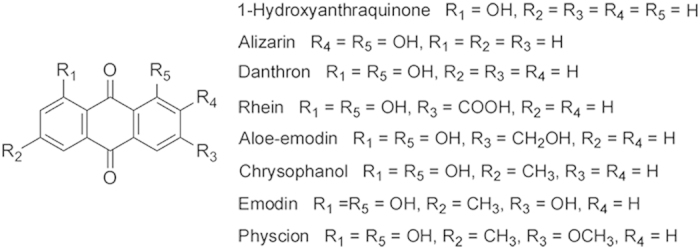
Chemical structures of eight monomeric anthraquinones.

**Table 1:** Blood clearance of ^131^I-rhein.

Parameter	Unit	Value for normal rats
AUC_(0 –t)_	MBq/L*h	187.46 ± 28.26
AUC_(0 –∞)_	MBq/L*h	211.76 ± 31.18
t_1/2z_	h	8.20 ± 0.49
T_max_	h	0.08 ± 0
CL_Z_	L/h/kg	0.04 ± 0.01
C_max_	MBq/L	42.40 ± 5.41
V_Z_	L/kg	0.42 ± 0.06

Major pharmacokinetics parameters of ^131^I-rhein derived by the statistical moment method of the non-compartment model in normal rats within 24 h after intravenous injection at a dose of 3.7 MBq each rat (n = 6). AUC_(0–t)_ and AUC_(0–∞)_: area under the curve; t_1/2z_: elimination half-life; T_max_: peak time; CL_Z_: clearance; C_max_: peak concentration. The values are expressed as mean ± SD.
