# Supplementary material for: Discovery of Radioiodinated Monomeric Anthraquinones as a Novel Class of Necrosis Avid Agents for Early Imaging of Necrotic Myocardium
Source: Sci Rep. 2016 Feb 16;6:21341. doi: 10.1038/srep21341 (PMC4754898; doi:10.1038/srep21341)
Supplement: Supplementary Information [file srep21341-s1.pdf]

Discovery of Radioiodinated Monomeric Anthraquinones as a Novel Class of Necrosis Avid Agents for Early Imaging of Necrotic Myocardium

Qin Wang[1,2]*, Shengwei Yang[1,2], Cuihua Jiang[1,2], Jindian Li[1,2,3], Cong Wang[1,2,3], Linwei Chen[1]*, Qiaomei Jin[1,2], Shaoli Song[4], Yuanbo Feng[1,2,5], Yicheng Ni[1,2,5], Jian Zhang[1,2] & Zhiqi Yin[3]

[1]Affiliated Hospital of Integrated Traditional Chinese and Western Medicine, Nanjing University of Chinese Medicine, Nanjing 210028, China, [2]Laboratories of Translational Medicine, Jiangsu Province Academy of Traditional Chinese Medicine, Nanjing 210028, China, [3]Department of Natural Medicinal Chemistry & National Center of Drug Screening, China Pharmaceutical University, Nanjing 210009, China, [4]Department of Nuclear Medicine, Renji Hospital, Shanghai Jiaotong University, School of Medicine, Shanghai 200127, China, [5]Theragnostic Laboratory, Campus Gasthuisberg, KU Leuven, 3000 Leuven, Belgium

Correspondence and requests for materials should be addressed to J.Z. (zhangjian@jsatcm.com) or Z.Y. (chyzq2005@126.com)

*These authors contributed equally to this work.

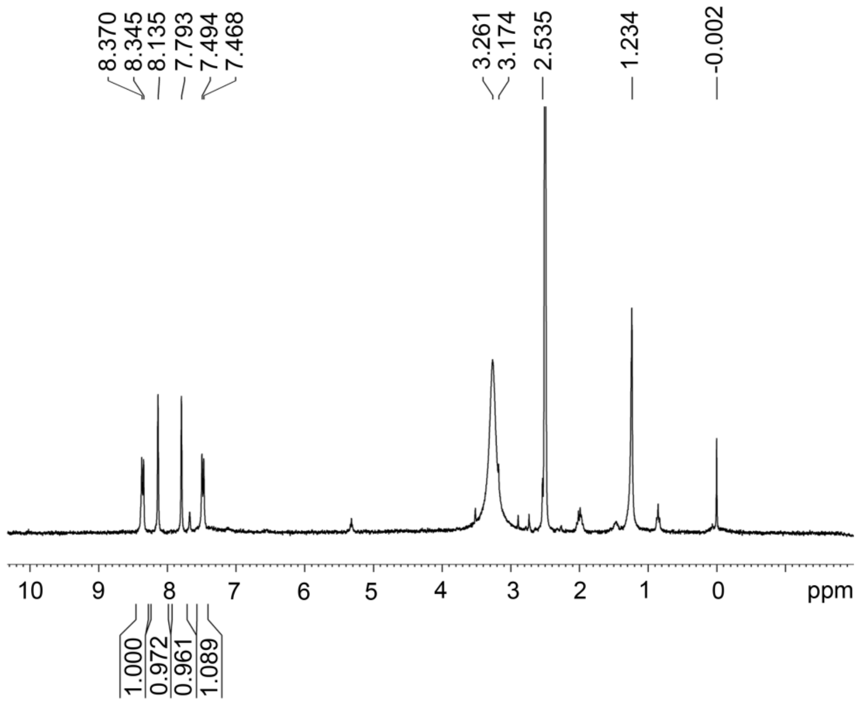

Supplementary Figure S1. ^{1}H NMR spectrum of mono-iodorhein.

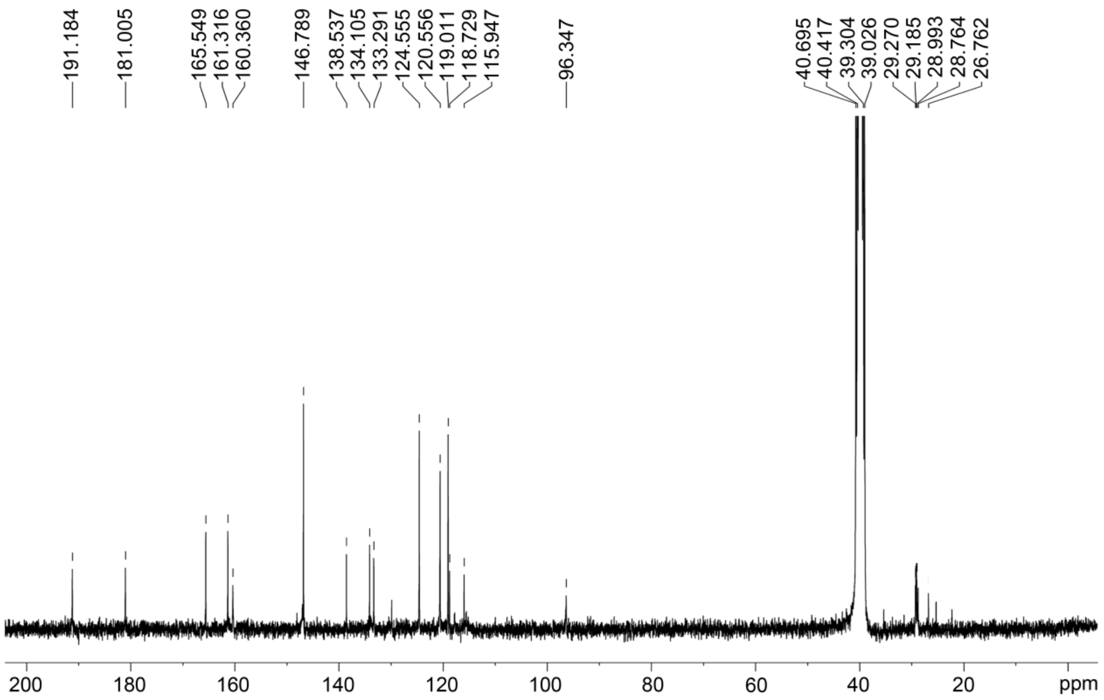

Supplementary Figure S2. ^{13}C NMR spectrum of mono-iodorhein.

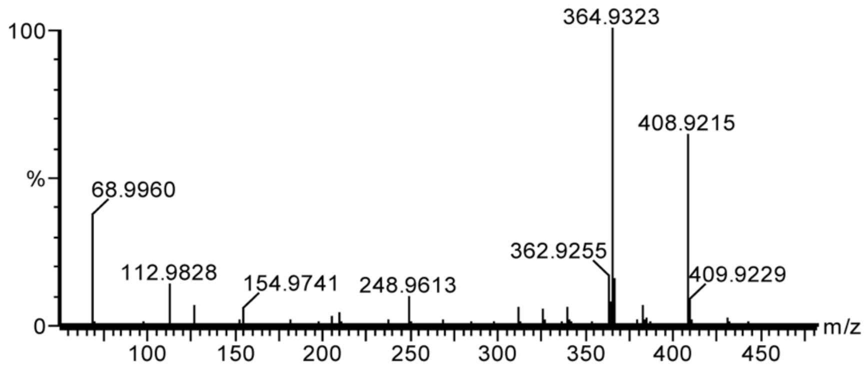

Supplementary Figure S3. HR-ESI-MS spectrum of mono-iodorhein.

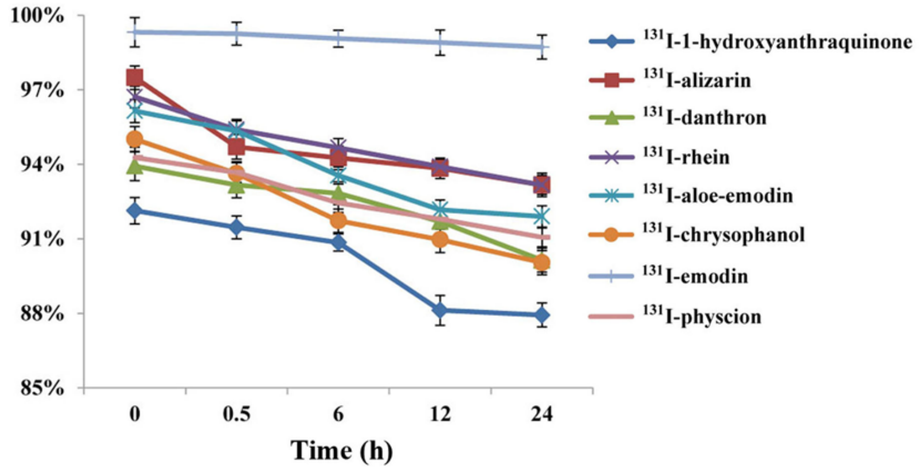

Supplementary Figure S4. Radiochemical purity and *in vitro* stability of eight ^{131}I-anthraquinones at different time points (n = 3) by TLC.

Supplementary Table S1. Biodistribution studies of ^{131}I-1-hydroxyanthraquinone, ^{131}I-alizarin, ^{131}I-danthron and ^{131}I-rhein. As measured by gamma counting in model mice at 2 h, 12 h and 24 h after administration (n = 5 per tracer). The injection dose of each mouse is 0.37 MBq. Data are expressed as average percentage of injected dose per gram (%ID/g) ± SD.

Organ	^{131}I-1-hydroxyanthraquinone			^{131}I-alizarin			^{131}I-danthron			^{131}I-rhein		
	2 h	12 h	24 h	2 h	12 h	24 h	2 h	12 h	24 h	2 h	12 h	24 h
Blood	3.19 ± 0.35	0.66 ± 0.04	0.07 ± 0.01	3.03 ± 0.42	0.74 ± 0.09	0.13 ± 0.01	2.71 ± 0.33	0.84 ± 0.09	0.31 ± 0.02	3.14 ± 0.32	1.20 ± 0.13	0.15 ± 0.01
Thyroid	1.53 ± 0.21	0.46 ± 0.03	0.33 ± 0.04	1.81 ± 0.29	0.61 ± 0.08	0.14 ± 0.03	1.85 ± 0.24	0.75 ± 0.06	0.31 ± 0.06	1.56 ± 0.17	0.78 ± 0.07	0.32 ± 0.06
Lung	1.12 ± 0.09	0.33 ± 0.04	0.05 ± 0.01	0.93 ± 0.08	0.25 ± 0.02	0.07 ± 0.01	1.10 ± 0.09	0.47 ± 0.02	0.23 ± 0.05	0.95 ± 0.06	0.62 ± 0.05	0.18 ± 0.03
Heart	0.58 ± 0.06	0.11 ± 0.01	0.02 ± 0.00	0.56 ± 0.07	0.20 ± 0.01	0.05 ± 0.00	0.50 ± 0.03	0.21 ± 0.02	0.08 ± 0.01	0.47 ± 0.05	0.28 ± 0.03	0.08 ± 0.01
Liver	1.07 ± 0.11	0.24 ± 0.03	0.08 ± 0.01	2.57 ± 0.23	1.15 ± 0.12	0.47 ± 0.05	2.81 ± 0.32	1.68 ± 0.15	1.07 ± 0.19	2.03 ± 0.21	1.55 ± 0.12	0.49 ± 0.05
Spleen	1.04 ± 0.08	0.26 ± 0.02	0.02 ± 0.00	0.97 ± 0.11	0.33 ± 0.04	0.13 ± 0.02	1.07 ± 0.08	0.60 ± 0.07	0.36 ± 0.04	0.97 ± 0.13	0.65 ± 0.08	0.29 ± 0.03
Stomach	1.40 ± 0.13	0.82 ± 0.09	0.09 ± 0.01	1.45 ± 0.17	0.71 ± 0.07	0.10 ± 0.02	1.21 ± 0.11	0.92 ± 0.08	0.20 ± 0.03	1.40 ± 0.16	1.05 ± 0.11	0.09 ± 0.02
Kidney	1.64 ± 0.17	0.37 ± 0.02	0.04 ± 0.01	2.85 ± 0.33	1.23 ± 0.11	0.60 ± 0.05	2.28 ± 0.34	1.96 ± 0.17	0.67 ± 0.08	2.68 ± 0.47	2.03 ± 0.12	0.65 ± 0.07
Viable.m	0.74 ± 0.05	0.14 ± 0.01	0.06 ± 0.00	0.61 ± 0.07	0.23 ± 0.02	0.03 ± 0.00	0.63 ± 0.07	0.35 ± 0.02	0.06 ± 0.01	0.79 ± 0.08	0.56 ± 0.04	0.06 ± 0.00
Necrotic.m	2.75 ±0.31	0.59 ± 0.05	0.35 ± 0.02	2.49 ± 0.29	0.98 ± 0.07	0.19 ± 0.01	2.59 ± 0.26	1.60 ± 0.07	0.40 ± 0.03	4.61 ± 0.39	4.06 ± 0.16	0.74 ± 0.04
N/V ratio	3.71 ± 0.37	4.22 ± 0.02	5.83 ± 0.29	4.08 ± 0.01	4.26 ± 0.07	6.33 ± 0.33	4.11 ± 0.04	4.57 ± 0.06	6.74 ± 0.63	5.84 ± 0.10	7.26 ± 0.23	12.33 ± 0.67

Viable.m = viable muscle, Necrotic.m = necrotic muscle, N/V = necrotic muscle /viable muscle.

Supplementary Table S2. Biodistribution studies of ^{131}I-aloe-emodin, ^{131}I-chrysophanol, ^{131}I-emodin and ^{131}I-physcion. As measured by gamma counting in model mice at 2 h, 12 h and 24 h after administration (n = 5 per tracer). The injection dose of each mouse is 0.37 MBq. Data are expressed as average percentage of injected dose per gram (%ID/g) ± SD.

Organ	^{131}I-aloe-emodin			^{131}I-chrysophanol			^{131}I-emodin			^{131}I-physcion		
	2 h	12 h	24 h	2 h	12 h	24 h	2 h	12 h	24 h	2 h	12 h	24 h
Blood	2.48 ±0.31	1.00 ±0.11	0.28 ±0.03	2.28 ±0.35	0.86 ±0.09	0.10 ±0.02	2.24 ±0.28	1.18 ±0.14	0.19 ±0.02	2.45 ±0.31	0.80 ±0.06	0.08 ±0.01
Thyroid	1.79 ±0.24	0.82 ±0.07	0.29 ±0.02	1.32 ±0.14	0.83 ±0.07	0.38 ±0.04	1.07 ±0.11	0.51 ±0.03	0.16 ±0.01	1.51 ±0.17	0.95 ±0.08	0.42 ±0.04
Lung	1.13 ±0.12	0.69 ±0.05	0.11 ±0.01	0.61 ±0.07	0.38 ±0.04	0.14 ±0.02	0.54 ±0.07	0.13 ±0.02	0.09 ±0.01	1.14 ±0.12	0.32 ±0.02	0.09 ±0.01
Heart	0.49 ±0.06	0.26 ±0.03	0.07 ±0.01	0.40 ±0.03	0.15 ±0.02	0.02 ±0.00	0.37 ±0.04	0.13 ±0.01	0.04 ±0.00	0.43 ±0.05	0.18 ±0.02	0.02 ±0.00
Liver	1.87 ±0.20	1.10 ±0.14	0.52 ±0.04	1.54 ±0.16	0.47 ±0.06	0.13 ±0.02	1.66 ±0.19	0.60 ±0.03	0.21 ±0.02	1.56 ±0.14	0.43 ±0.05	0.08 ±0.01
Spleen	1.14 ±0.18	0.55 ±0.05	0.16 ±0.01	0.73 ±0.09	0.24 ±0.03	0.07 ±0.01	0.87 ±0.06	0.42 ±0.05	0.09 ±0.01	0.92 ±0.08	0.34 ±0.04	0.04 ±0.00
Stomach	1.63 ±0.11	1.15 ±0.03	0.20 ±0.02	1.83 ±0.21	1.23 ±0.09	0.08 ±0.01	1.41 ±0.13	1.26 ±0.10	0.27 ±0.03	1.79 ±0.16	1.10 ±0.12	0.09 ±0.02
Kidney	2.72 ±0.34	2.04 ±0.16	0.68 ±0.03	1.86 ±0.17	0.64 ±0.07	0.10 ±0.01	1.55 ±0.18	0.74 ±0.06	0.16 ±0.01	1.27 ±0.13	0.56 ±0.03	0.09 ±0.01
Viable.m	0.31 ±0.03	0.17 ±0.02	0.06 ±0.00	0.39 ±0.05	0.26 ±0.03	0.02 ±0.00	0.40 ±0.05	0.14 ±0.02	0.03 ±0.00	0.36 ±0.04	0.19 ±0.02	0.02 ±0.00
Necrotic.m	1.43 ±0.17	0.95 ±0.07	0.47 ±0.04	1.73 ±0.23	1.25 ±0.09	0.14 ±0.01	1.52 ±0.13	0.60 ±0.07	0.18 ±0.02	1.13 ±0.17	0.71 ±0.06	0.10 ±0.01
N/V ratio	4.52 ±0.12	5.61 ±0.25	7.83 ±0.67	4.43 ±0.02	4.82 ±0.21	7.01 ±0.49	3.81 ±0.15	4.30 ±0.11	6.02 ±0.58	3.13 ±0.13	3.74 ±0.08	5.01 ±0.49

Viable.m = viable muscle, Necrotic.m = necrotic muscle, N/V = necrotic muscle/ viable muscle.
